# Enhancing Healthcare Access for Older Adults through Innovative Mobility Solutions Leveraging Public Assets

**DOI:** 10.31662/jmaj.2024-0358

**Published:** 2025-10-03

**Authors:** Mari Yokota, Shinji Nakahara, Masamichi Nishida

**Affiliations:** 1 Department of Emergency and General Medicine, Tokyo Teishin Hospital, Tokyo, Japan

**Keywords:** postal network, mobility service, older adults, healthcare, home-visit

## Abstract

Japan’s aging population and declining public transportation have reduced healthcare access, particularly for older adults in rural areas. Currently, innovative mobility services such as ridesharing, mixed cargo/passenger transport, and medical “Mobility as a Service” are being implemented or piloted alongside existing services for older adults, such as home-visit medical care and transportation subsidies. This letter highlights ongoing initiatives in Japan and their potential to improve healthcare access. It also explores opportunities to enhance these efforts by leveraging public infrastructure, such as the nationwide post office network.

## Introduction

Japan’s rapidly aging population is increasingly struggling to access medical services, particularly in rural areas with limited public transportation options ^(1)^. In 2020, 29% of Japan’s population was aged ≥65 years, projected to reach 39% by 2070 ^(2)^. While individuals with cars can still access services despite limited public transportation, older drivers are under social pressure to stop driving due to concerns over age-related car crashes, leaving many older adults―especially those living alone―reliant on public transportation ^(1)^.

Declining public transportation in rural areas exacerbates this issue ^(1)^. Preference for private cars has reduced public transit use and financially strained transportation services. Labor shortages due to a shrinking young workforce and working-hour reforms have further impaired service availability. Support measures such as municipal transportation and home-visit medical care are challenging to sustain in sparsely populated areas due to operational burdens.

Japan has largely addressed barriers to healthcare access, including economic constraints, uneven resource distribution, and systemic issues, through universal health coverage, abundant medical facilities, and free provider choices, leading to substantial health gains in the past several decades ^(3), (4)^. However, transportation has become a significant access issue for older individuals with limited mobility options. Restricted access would delay or limit care, potentially compromising disease management, increasing complications, undermining preventive efforts, and ultimately reversing these health gains.

A potential solution is “Mobility as a Service” (MaaS), which combines various transport modes and services into a single platform accessible through digital technologies ^(1)^. Some MaaS models divert cargo trucks to passenger transport or transport the service itself closer to people. Since 2018, Japan has piloted localized MaaS programs, including medical MaaS with mobile clinics and telemedicine. However, challenges remain, including older adults’ unfamiliarity with digital devices and privacy concerns that limit online medical care in public facilities.

Medical MaaS can enhance older adults’ access to healthcare in remote areas without overburdening medical professionals. The better use of public assets, including facilities and personnel, may help address these challenges. This paper reviews the current efforts to improve healthcare access for older adults, focusing on MaaS projects and other transport-based solutions. It also explores potential improvements in healthcare access, particularly by leveraging existing public assets and expanding services to bridge mobility gaps.

## Existing Measures

Longstanding efforts have been implemented to improve access to healthcare, both traditional and innovative. From a transportation perspective, services can be divided into two categories: transporting individuals to service venues and delivering services directly to individuals ^(1)^. The first category includes subsidies for public transport, and on-demand shared transport, such as public rideshare programs, private ridesharing, and mixed cargo/passenger transport. The second category includes home-visit medical care and medical MaaS. As automated driving, while promising, is not yet ready for large-scale use, we exclude its discussion here.

### Transporting Individuals to Service Venues

#### Subsidies for transport services

Many municipalities subsidize public transportation for older adults, offering discounted or free vouchers for trains, buses, and taxis to enhance their mobility. However, these efforts are less effective in areas where public transportation infrastructure is declining.

#### On-demand transport

On-demand transport services, which provide door-to-door transport based on reservations, are either run directly by municipalities or subsidized. These services are more convenient for users than fixed-route options and are more cost-efficient for providers ^(1)^. However, one drawback is that app-based reservations generally must be made at least 1 day in advance.

### Ridesharing

Ridesharing in Japan has been restricted due to strict regulations on transport services. Although “public ridesharing” services, introduced in 2006, should be managed by municipalities or non-profit organizations and are limited to areas with no public transportation or individuals with disabilities, this offers limited benefits for older adults without disabilities.

In March 2024, ridesharing by non-commercial drivers using private vehicles was approved; however, coverage remains limited to urban areas. This service is only permitted when and where taxi demand exceeds supply, based on data from taxi dispatch apps available only in urban areas.

### Mixed cargo/Passenger transport

Since 2023, cargo transporters have been allowed to carry both goods and passengers. This initiative leverages existing logistics networks to help meet the transportation needs of older adults in rural areas while minimizing costs.

For example, postal delivery vehicles can transport other goods and passengers by utilizing available space. Users can reserve rides via an app-based system and meet the vehicle at a designated post box for travel to a post office or another stop along a set route ^(5)^. These vehicles also deliver online orders to designated community locations, improving shopping access and fostering community interaction ^(6)^.

### Bringing Services to Individuals

#### Home-visit medical care

Home-visit medical care involves physicians visiting patients’ residences. This service includes scheduled visits, often multiple times per month, as well as emergency visits for sudden health issues. The number of patients receiving scheduled visits is increasing, with 90% being older adults aged ≥75 years. These services are primarily provided by private clinics in areas with aging populations and limited healthcare access. However, in sparsely populated regions, long distances between patients’ homes can strain healthcare providers.

### Medical MaaS

Medical MaaS enhances healthcare access by offering flexible, responsive mobility solutions that reduce the burden on both patients and healthcare providers ^(1)^. Whereas the typical medical MaaS model brings diagnostic or treatment equipment and facilities closer to patients, another model helps patients travel to medical facilities. This section focuses on the first model, as the second primarily integrates medical consultation reservations into existing on-demand/shared transport services.

The medical MaaS model includes mobile medical vehicles where nurses facilitate online consultations, providing services in communities and easing travel burdens for doctors and patients in sparsely populated areas. Individuals unfamiliar with digital technology can still receive care with support from onboard nurses. Collaborative efforts have enabled mobile medical vehicles to provide services at community centers, with assistance from center staff.

Alternatively, online medical consultations can be provided at public facilities that ensure privacy or directly at home. Some post offices have piloted these services at their locations or patients’ homes, with postal staff assisting with device use and direct prescription delivery to homes ^(7), (8)^. When receiving consultations at home, post offices provide a home-visit service with a medical tablet. However, the availability of these services remains limited.

## Discussion

Various novel measures are being implemented or piloted to enhance mobility and improve healthcare access for older adults, particularly by integrating innovations into existing infrastructure. For instance, mixed cargo/passenger transport leverages established logistics and digital reservation systems, while medical MaaS combines public facilities with online services. However, many older adults face challenges with technologies such as reservation and communication apps due to limited familiarity with digital interfaces.

Studies in digitally advanced countries highlight the necessity of supporting older individuals in using digital systems. In the United States (US), rideshare programs with reservation assistance―by phone or through medical staff―have improved treatment adherence and increased healthcare utilization among Medicaid beneficiaries, patients undergoing colonoscopy, and individuals requiring regular cancer care ^(9), (10), (11)^. Similarly, a US study among veterans and an Australian study among the general population show that most remote consultations occur by phone rather than video ^(12), (13)^. A US table distribution program demonstrated that most older recipients did not use the devices for video consultations ^(14)^.

Public assets, including facilities and personnel, can play crucial roles in mitigating this issue. Japan’s extensive public facilities―including municipal offices, community centers, libraries, and post offices― not only offer physical spaces for services but also provide staff to assist older adults navigate these systems. For example, public facilities can serve as convenient starting and ending points for mobility services, with staff assisting with reservations. Simpler options, such as phone-based ride-booking or receiving help from public facility staff for online consultations, are available to ensure accessibility to modern services for older adults. Although such assistance increases operating costs, public organizations are well-positioned to absorb these expenses.

Particularly, post office networks with over 24,000 office locations and extensive delivery systems stand out in terms of size and reach ^(15)^, offering greater accessibility than other public facilities. Despite semi-privatization in 2007, post offices remain publicly regulated and continue to offer public services. Hosting medical MaaS at post offices, which is still in the pilot stage, can further help expand medical MaaS nationwide. Additionally, expanded use of postal delivery vehicles for passenger transport can help older patients reach medical facilities, with postal staff assisting them with digital technologies.

In rural areas, where home-visit services are accepted, combining them with online medical care at home is an effective strategy, even if the visits do not directly deliver medical care. For example, post offices offer the “Mimamori” (monitoring) service, where postal staff regularly check on older adults and report their condition to family members ^(7)^. This service can easily be integrated with at-home online medical consultations. Trust in visitors is the key to the acceptance of home-visit services, and public facility staff are likely to be welcomed for such services. Post office staff, historically trusted for services such as home cash transactions, are well-positioned to expand these offerings.

Initiatives involving volunteers who monitor older adults are promising for improving healthcare access in communities, even if they do not directly provide medical treatment. During home visits, volunteers may help older adults access modern services through digital devices. One such initiative is the Dementia Supporter initiative, in which volunteers are trained to assist patients with dementia and their families. These volunteers include citizens and public sector employees, including municipal officers and postal staff, with public workers playing a significant role in-home visits. This workforce resource can contribute significantly to fostering a more supportive community.

### Conclusions

Access to medical care for older adults remains a challenge in regions with declining public transportation. Innovative mobility services, including medical MaaS, can improve healthcare access. This approach could be further enhanced by fully leveraging public assets, particularly for tech-averse older adults who require assistance with service use.

## Article Information

### Author Contributions

Mari Yokota: conceptualization, investigation, writing-original draft, writing-review & editing; Shinji Nakahara: conceptualization, investigation, writing-review & editing, fund acquisition; Masamichi Nishida: conceptualization, investigation, writing-review & editing, supervision.

### Conflicts of Interest

The authors are employed by Tokyo Teishih Hospital which is affiliated with Japan Post Co., Ltd. This paper represents the authors’ personal views. The authors are employees of Japan Post; however, the company had no involvement in the preparation of this manuscript.

### Ethical Approval

Not required because this is an opinion piece.
